# Travelers’ Actual and Subjective Knowledge about Risk for Ebola Virus Disease

**DOI:** 10.3201/eid2409.171343

**Published:** 2018-09

**Authors:** Isabelle Régner, Oana Elena Ianos, Loucy Shajrawi, Philippe Brouqui, Philippe Gautret

**Affiliations:** Aix-Marseille Université, Marseille, France (I. Régner);; Méditerranée Infection Institute, Marseille (O.E. Ianos, L. Shajrawi, P. Brouqui, P. Gautret)

**Keywords:** Ebola virus disease, risk perceptions, travelers, actual knowledge, subjective knowledge, viruses, France, Ebola, Ebola virus

## Abstract

To determine travelers’ actual and subjective knowledge about risk for Ebola virus disease, we surveyed travelers from France. Actual knowledge did not prevent irrational perceptions or promote safe behavior. Rather, readiness to adopt protective behavior depended on subjective knowledge and overconfidence in ability to self-protect.

The 2014–2016 epidemic of Ebola virus disease (EVD) in West Africa was the largest ever recorded. As for many other infectious diseases (*1**,**2*), surveys of knowledge, attitudes, and practices report suboptimal knowledge and misperceptions of risk for EVD among various populations (*3**–**6*). Recommendations typically emphasize the need to increase actual knowledge (what persons really know) to reduce irrational beliefs and risky behavior. However, subjective knowledge (what persons think they know), which has been overlooked in EVD surveys, can lead to the erroneous feeling that one has the requisite knowledge to avoid adverse events, resulting in a higher risk of experiencing negative outcomes (*7*). To determine if actual and subjective knowledge about EVD would lead to differing perceptions of risk, we surveyed travelers from France who had visited the International Vaccination Center at North Hospital in Marseille, France, for pretravel consultation during May 2015–February 2016. 

A sample of 189 participants (93 women, 96 men; mean age ± SD 37.78 ± 14.50 years) anonymously completed a questionnaire about their knowledge and perceptions of risk of acquiring EVD. Respondents reported their sociodemographic characteristics, destination, purpose of travel, date of departure, and date of return. Questions about EVD actual knowledge included preventive measures, transmission routes, epidemic status, affected countries, and presence of EVD in the destination country. We used correct responses to compute final scores (Technical Appendix). We used 5-point Likert scales (1 = strongly disagree to 5 = strongly agree) to record travelers’ self-reports pertaining to their subjective knowledge (*7*) and several risk perceptions about EVD (*6**,**8**,**9*): perceived seriousness of EVD, awareness of EVD risk in the destination country, perceived effectiveness of protective measures, fear of contracting EVD in the country of destination, fear of contracting EVD in Europe, and intentions to adopt preventive behavior. Personal control and unrealistic optimism were assessed as key measures of positive illusions that typically lead persons to overestimate their capabilities to protect themselves against adverse events (*8**,**9*) (Technical Appendix).

Among the 189 participants, 25.9% planned to travel to West Africa (2.6% to an affected country, Guinea), 21.7% to other African countries, and 52.4% to other countries worldwide. Only 10.6% were able to correctly report the 3 countries affected by the EVD epidemic (Liberia, Sierra Leone, Guinea), and many were unaware of preventive measures (45%) and modes of Ebola virus transmission (39.1%). The most frequent answers for preventive measures were practice careful hygiene (24.34%), avoid contact with infected persons (23.28%), and wear protective equipment (21.16%). Answers about modes of Ebola virus transmission were body contact (31.22%), body fluids (30.16%), and aerosol (12.17%; this answer is wrong). Overall, the actual knowledge about EVD was very low (mean 3.57 correct responses; maximum possible score = 16). Simultaneously, subjective knowledge was low (mean ± SD 2.39 ± 1.00; maximum possible score = 5.00) (Technical Appendix Table 3 for bivariate intercorrelations).

To go beyond bivariate correlations and to estimate the associations between risk perceptions and each type of knowledge, we used multiple regression analyses (Table). Findings showed that actual knowledge was far from being as effective, as typically thought from knowledge, attitudes, and practices studies (*3**–**5*). Actual knowledge was associated only with higher perceived seriousness of the disease and lower awareness of risk for EVD in the country of destination, which reflects some rational perceptions (EVD is indeed serious, and most destination countries for this sample population were not affected by the epidemic). However, travelers with greater actual knowledge were not more likely to view protective measures as efficient, to avoid positive illusions, or to intend to engage in protective behavior. On the contrary, travelers with higher subjective knowledge reported confidence in preventive measures and intention to adopt safe behavior, while indicating illusions of having personal control and unrealistic optimism. Results of a further analysis (Technical Appendix) revealed that positive illusions and subjective knowledge were positively associated with behavioral intentions.

**Table:** Results of multiple regression analyses for variables predicting actual and subjective knowledge of risk for Ebola virus disease*

Risk perception variable	Actual knowledge			Subjective knowledge	
	*b*	95% CI		*b*	95% CI
Perceived seriousness	0.12 (p<0.001)	0.05 to 0.20		0.08	–0.08 to 0.24
Risk awareness	–0.19 (p<0.001)	–0.26 to –0.11		–0.16 (p<0.05)	–0.33 to –0.01
Perceived effectiveness of protective measures	0.04	–0.02 to 0.10		0.22 p<0.01)	0.09 to 0.35
Positive illusions	–0.07	–0.14 to 0.01		0.16 (p<0.05)	0.01 to 0.33
Fear of contracting EVD in destination country	0.03	–0.07 to 0.12		0.02	–0.19 to 0.22
Fear of contracting EVD in Europe	–0.01	–0.09 to 0.07		-0.09	–0.26 to 0.08
Behavioral intention	0.04	–0.03 to 0.11		0.16 (p<0.05)	0.01 to 0.32
% variance explained by the model	Adj R^2^ = 0.32 (p<0.001)			Adj R^2^ = 0.21 (p<0.001)	

*All regression coefficients are unstandardized coefficients that were adjusted for participants’ destination (Africa vs. other countries). Adj, adjusted; *b*, unstandardized regression coefficients; EVD, Ebola virus disease.

Our observations of suboptimal actual knowledge about EVD replicated findings of past knowledge, attitudes, and practices studies (*3**–**6*); however, we went further by showing that relationships between actual versus subjective knowledge and perceptions of risk for EVD differed. The fact that subjective knowledge and positive illusions, but not actual knowledge, were associated with protective behavior intentions is problematic, especially because actual knowledge was low. Persons’ belief that they know how to protect themselves when they actually do not and the feeling of knowing added to a feeling of overconfidence in how to self-protect might result in risky rather than safe behavior (*7*). 

Our results indicate that not considering subjective knowledge and positive illusions can lead to the erroneous conclusion that increasing actual knowledge will necessarily translate into behavioral change and good practices. EVD communication would benefit from research showing that promoting behavioral change requires changing subjective evaluations of risk to make it self-relevant and to induce a reappraisal of the perceived benefits of (or costs of not) performing safe behavior (*10*).

Technical Appendix Additional methods and results for study of travelers’ actual and subjective knowledge about risk for Ebola virus disease.

## References

[1] Sridhar S, Belhouchat K, Drali T, Benkouiten S, Parola P, Brouqui P, et al. French Hajj pilgrims’ experience with pneumococcal infection and vaccination: A knowledge, attitudes and practice (KAP) evaluation. Travel Med Infect Dis. 2015;13:251–5. 10.1016/j.tmaid.2015.02.002 25725996 PMC7110623

[2] Tashani M, Alfelali M, Barasheed O, Fatema FN, Alqahtani A, Rashid H, et al. Australian Hajj pilgrims’ knowledge about MERS-CoV and other respiratory infections. Virol Sin. 2014;29:318–20. 10.1007/s12250-014-3506-y 25338843 PMC7091107

[3] Rübsamen N, Castell S, Horn J, Karch A, Ott JJ, Raupach-Rosin H, et al. Ebola risk perception in Germany, 2014. Emerg Infect Dis. 2015;21:1012–8. 10.3201/eid2106.150013 25989020 PMC4451905

[4] Alqahtani AS, Wiley KE, Willaby HW, BinDhim NF, Tashani M, Heywood AE, et al. Australian Hajj pilgrims’ knowledge, attitude and perception about Ebola, November 2014 to February 2015. Euro Surveill. 2015;20:21072. 10.2807/1560-7917.ES2015.20.12.21072 25846489

[5] Merino JG. Response to Ebola in the US: misinformation, fear, and new opportunities. BMJ. 2014;349(nov07 13):g6712. 10.1136/bmj.g6712 25380659

[6] Sridhar S, Brouqui P, Fontaine J, Perivier I, Ruscassier P, Gautret P, et al. Risk perceptions of MSF healthcare workers on the recent Ebola epidemic in West Africa. New Microbes New Infect. 2016;12:61–8. 10.1016/j.nmni.2016.04.010 27330816 PMC4900694

[7] Jaccard J, Dodge T, Guilamo-Ramos V. Metacognition, risk behavior, and risk outcomes: the role of perceived intelligence and perceived knowledge. Health Psychol. 2005;24:161–70. 10.1037/0278-6133.24.2.161 15755230

[8] Schneider SL. In search of realistic optimism. Meaning, knowledge, and warm fuzziness. Am Psychol. 2001;56:250–63. 10.1037/0003-066X.56.3.250 11315251

[9] Peterson C, Stunkard AJ. Personal control and health promotion. Soc Sci Med. 1989;28:819–28. 10.1016/0277-9536(89)90111-1 2649994

[10] Dijkstra A, Rothman A. Social psychology of health and illness. In: Steg L, Keizer K, Buunk AP, Rothengatter T, editors. Applied Social Psychology. Cambridge (UK): Cambridge University Press; 2017. p. 214–34.

