# Supplementary material for: Travelers’ Actual and Subjective Knowledge about Risk for Ebola Virus Disease
Source: Emerg Infect Dis. 2018 Sep;24(9):1750–1. doi: 10.3201/eid2409.171343 (PMC6106432; doi:10.3201/eid2409.171343)
Supplement: Technical Appendix — Additional methods and results for study of travelers’ actual and subjective knowledge about risk for Ebola virus disease. [file 17-1343-Techapp-s1.pdf]

Article DOI: https://doi.org/10.3201/eid2409.171343

Travelers' Actual and Subjective Knowledge about Risk for Ebola Virus Disease

Technical Appendix

Participants

Between May 2015 and February 2016, around 3000 travelers visited the International Vaccination Center at North Hospital in Marseille (France). Among them, 20% were children or adolescents and 13.5% were pilgrims (who were treated in a specific campaign), which left around 2000 travelers eligible for the survey. For practical reasons within the Vaccination Center, participation to the survey was systematically proposed to eligible travelers only 1 day per week, which resulted in a potential sample of 392 travelers. Among them, 253 travelers agreed to complete the questionnaire. However, 25.30% of them did not respond to either knowledge or risk perception questions and were excluded from the study, resulting in a sample of 189 travelers.

Technical Appendix Table 1. Demographic characteristics of the 189 participants

Characteristics	n (%)
Sex	
M	96 (50.8)
F	93 (49.2)
Mean Age	37.78 y (SD = 14.50, Min 18, Max 71)
Education level	
None	33 (17.5)
Secondary level	52 (27.5)
Bachelor degree or higher	102 (54)
Missing information	2 (1)
Occupations	
Farmer	1 (0.5)
Craftman, Shopkeeper, Business owner	6 (3.2)
Executives and upper intellectual occupations	35 (18.5)
Health and social workers, school teachers	38 (20.1)
Employees	22 (11.6)
Workers	8 (4.2)
Retired	16 (8.5)
Unemployed, non-working	56 (29.6)
Missing information	7 (3.7)
Country of birth	
France	152 (80.4)
West Africa	10 (5.3)
Other African countries	17 (9)
Others	10 (5.3)

Technical Appendix Table 2. Actual knowledge scores assigned to each response for all categories of questions

Affected countries	Score
Guinea	1
Sierra Leone	1
Liberia	1
Nigeria	0.5
Senegal	0.5
Mali	0.50
Don’t know	0
Other countries in Africa	0
All other countries	0
Presence of EVD in the destination country	
Right answer	1
False answer	0
Don’t know	0
Epidemic status	
Still ongoing and severe	0.5
Still ongoing but less severe	1
The epidemic is over	0
Don’t know	0
Transmission routes	
Animals-to-human transmission	1
Contact with infected dead bodies animals/human	1
Sexual transmission	1
Infected nutrition/ eating bush meat	1
Body fluids	1
Aerosol transmission	0
Insects	0
Don’t know	
Preventive measures	
Avoid contacts with animals (bats) and patients/corpse	1
Using protective equipment during contact such as gloves and condom	1
Cooking the meat	1
Hygiene - wash hands	1
Don’t know	0
Maximum possible score	16

Risk perception variables

Subjective knowledge about EVD was assessed using three items adapted from Jaccard et al. (*1*): “I think I have enough knowledge about EVD,” “I know well the preventive measures against EVD,” “I have a good knowledge of the EVD transmission routes.” Responses were obtained on a 5-point Likert scale (from 1 = *strongly disagree* to 5 = *strongly agree*) and were averaged (Cronbach's α = 0.84).

Based on previous research on risk perceptions (*2–4*), participants rated several items using 5-point Likert scale (from 1 = *strongly disagree* to 5 = *strongly agree*) designed to estimate risk perception variables. Participants were asked to report their perceived seriousness of EVD: “*Ebola is easily transmitted*,” “*The cure rate of patients infected with Ebola is low*.” Two items measured their risk awareness of EVD in the country of destination: “*My destination country is at risk for Ebola*,” “*During my stay, the risk of contracting Ebola is less important than the risk*

of contracting other diseases" (reverse coded). Participants indicated to what extent they considered that the protective measures against Ebola were efficient: "*Protective measures against Ebola are efficient*." They were also asked to report on their fear for contracting EVD in the country of destination ("*I'm afraid of contracting Ebola during my stay*," "*I'm worried about the possibility of being contaminated in the airplane by an infected passenger*") as well as in Europe ("*I'm worried about the possibility of an Ebola epidemic in Europe*," "*I'm afraid of contracting Ebola in France").* Intentions to adopt preventive behaviors against Ebola were measured by 3 items: "*Because of the epidemic of Ebola, I will be more careful than usual during my stay*," "*The Ebola epidemic has changed my plans during my stay*," "*Due to the Ebola epidemic, I will limit contacts with the local population*." Two items captured participants' unrealistic optimism: "*I think I have less risk of contracting the Ebola virus than the local population*," "*If I was infected with the Ebola virus, I think I would have a better chance of recovering than other people of the same age and gender*." These items were reversed to compute an average score so that higher scores would indicate higher unrealistic optimism. Finally, participants also reported on their personal control: "*If I was exposed to the Ebola virus, I think I would have the ability to avoid being contaminated*," "*I think I'm healthy enough to avoid infection with the Ebola virus*."

Descriptive statistics and bivariate correlations

Technical Appendix Table 3. Means, standard deviations, and Pearson's bivariate correlations

Variables	Means (SD)	Possible range scores	1	2	3	4	5	6	7	8	9	10
1-Actual knowledge	3.57 (2.37)	0–16	–									
2-Subjective knowledge	2.39 (0.99)	1–5	.52***	–								
3-Perceived seriousness	3.48 (0.89)	1–5	.35***	.20**	–							
4-Risk awareness	2.34 (0.99)	1–5	-.28***	-.09	-.05	–						
5-Perceived efficacy of protective measures	3.01 (1.04)	1–5	.18*	.30***	.14	.04	–					
6-Personal control	2.16 (1.04)	1–5	.06	.32***	-.02	.08	.21**	–				
7-Unrealistic optimism	2.01 (1.02)	1–5	.06	.23*	.01	.00	.19*	.52***	–			
8-Fear for contacting EVD in the country of destination	1.87 (0.82)	1–5	-.04	.01	.17*	.39***	.08	.03	-.02	–		
9-Fear for contracting EVD in Europe	1.99 (0.88)	1–5	-.08	-.05	.04	.10	.07	-.02	-.12	.35***	–	
10-Behavioral intentions	2.45 (1.08)	1–5	.14*	.35***	.22**	.31***	.21**	.31***	.20**	.36***	.24***	–

* $p < 0.05$, ** $p < 0.01$, *** $p < 0.001$.

Analytical strategy

To compare the association between actual knowledge and subjective knowledge about EVD with several risk perceptions, our first strategy was to enter both types of knowledge as predictors in multiple regression analyses with each risk perception variable as the outcome. However, as shown in Table 3, bivariate correlations revealed that actual and subjective knowledge were highly correlated (r = 0.52). Although this positive and significant correlation is not surprising (*1*), its size could raise a multicollinearity concern. For that reason, we rather treated both types of knowledge as an outcome and used all the other risk perception variables as predictors. It is noteworthy that both analytical strategies led to the same key findings.

Likewise, because personal control and unrealistic optimism were highly correlated (r = 0.52), these variables were averaged to reflect positive illusions and entered as such in the regression models.

A complementary analysis was conducted to estimate the respective contribution of positive illusions and subjective knowledge to behavioral intentions. For that purpose, we regressed behavioral intentions on both variables (while controlling for the other risk perceptions). Results are described in Table 4 below.

Technical Appendix Table 4. Summary of multiple regression analysis for risk perceptions variables predicting behavioral intentions

	Behavioral Intentions	
Risk perception variables	*b*	95% CI
Perceived seriousness	.23**	.09,.38
Risk awareness	.13	-.02,.29
Perceived efficacy of protective measures	.09	-.05,.22
Positive illusions	.22**	.06,.38
Fear for contacting EVD in the country of destination	.24*	.05,.44
Fear for contracting EVD in Europe	.21*	.04,.37
Subjective Knowledge	.16*	.01,.31
% of the variance explained by the model	$AdjR^2$ = 0.38***	

* p < 0.05, ** p < 0.01, *** p < 0.001. All regression coefficients are unstandardized coefficients that were adjusted for participants' destination (African countries *versus* other countries in the world).

Results indicated that positive illusions (b = 0.22, p = 0.008) and subjective knowledge (b = 0.16, p = 0.046) were both positively associated with behavioral intentions ($AdjR^2$ = 0.38, p < 0.001). In other words, participants seem to be willing to engage in protective behaviors when they think they are knowledgeable enough and when they overestimate their capabilities to protect themselves against EVD.

Limitations

The present study is correlational, which prevents any causal inference. Another limitation could be that most our respondents planned to travel to countries not affected by EVD. The findings could thus not generalize to those traveling to affected countries. One might indeed reason that travelers who plan to travel to an affected country would be more concerned by EVD and thus display higher levels of actual knowledge. However, even higher levels of actual knowledge and/or greater relevance of the situation are unlikely to change the overall pattern of results for several reasons. First, controlling for the destination country did not change the present results. Second, given the independent effects of actual and subjective knowledge showed here and in previous research (*1*), it is quite unlikely that higher levels of actual knowledge would change the overall pattern of results. Third, previous research (*2*) has shown that even highly concerned individuals like healthcare workers traveling to Ebola camps in Africa, who indeed displayed higher (average) levels of actual knowledge, were not immune to risk misperceptions: they underestimated their likelihood for contracting Ebola compared to their colleagues and, despite their high-risk status, they showed little concern about contracting Ebola during their mission. Finally, it is still informative to discover that despite intensive media coverage of the epidemic (as it was the case in France and other non affected countries), many people display a lack of knowledge and misperceptions about EVD (*5*), which could be important factors of dissemination in case of imported disease.

References

1. Jaccard J, Dodge T, Guilamo-Ramos V. Metacognition, risk behavior, and risk outcomes: the role of perceived intelligence and perceived knowledge. Health Psychol. 2005;24:161–70. PubMed http://dx.doi.org/10.1037/0278-6133.24.2.161

2. Sridhar S, Brouqui P, Fontaine J, Perivier I, Ruscassier P, Gautret P, et al. Risk perceptions of MSF healthcare workers on the recent Ebola epidemic in West Africa. New Microbes New Infect. 2016;12:61–8. PubMed http://dx.doi.org/10.1016/j.nmni.2016.04.010

3. Schneider SL. In search of realistic optimism. Meaning, knowledge, and warm fuzziness. Am Psychol. 2001;56:250–63. PubMed http://dx.doi.org/10.1037/0003-066X.56.3.250

4. Peterson C, Stunkard AJ. Personal control and health promotion. Soc Sci Med. 1989;28:819–28. PubMed http://dx.doi.org/10.1016/0277-9536(89)90111-1

5. Rübsamen N, Castell S, Horn J, Karch A, Ott JJ, Raupach-Rosin H, et al. Ebola risk perception in Germany, 2014. Emerg Infect Dis. 2015;21:1012–8. PubMed http://dx.doi.org/10.3201/eid2106.150013
